# Impact of monoolein on aquaporin1-based supported lipid bilayer membranes

**DOI:** 10.1088/1468-6996/16/4/045005

**Published:** 2015-08-13

**Authors:** Zhining Wang, Xida Wang, Wande Ding, Miaoqi Wang, Xin Qi, Congjie Gao

**Affiliations:** 1 Key Laboratory of Marine Chemistry Theory and Technology, Ministry of Education, Ocean University of China, Qingdao 266100, People’s Republic of China; 2 Key Laboratory of Marine Drugs, Ministry of Education, School of Medicine and Pharmacy, Ocean University of China, Qingdao 266003, People’s Republic of China

**Keywords:** aquaporin, monoolein, lipid mobility, biomimetic membrane, water purification

## Abstract

Aquaporin (AQP) based biomimetic membranes have attracted considerable attention for their potential water purification applications. In this paper, AQP1 incorporated biomimetic membranes were prepared and characterized. The morphology and structure of the biomimetic membranes were characterized by *in situ* atomic force microscopy (AFM), infrared absorption spectroscopy, fluorescence microscopy, and contact angle measurements. The nanofiltration performance of the AQP1 incorporated membranes was investigated at 4 bar by using 2 g l^−1^ NaCl as feed solution. Lipid mobility plays an important role in the performance of the AQP1 incorporated supported lipid bilayer (SLB) membranes. We demonstrated that the lipid mobility is successfully tuned by the addition of monoolein (MO). Through *in situ* AFM and fluorescence recovery after photo-bleaching (FRAP) measurements, the membrane morphology and the molecular mobility were studied. The lipid mobility increased in the sequence DPPC < DPPC/MO (*R*_MO_ = 5/5) < DOPC/MO (*R*_MO_ = 5/5) < DOPC, which is consistent with the flux increment and salt rejection. This study may provide some useful insights for improving the water purification performance of biomimetic membranes.

## 1. Introduction

The lack of clean water resources has forced us to develop advanced water purification technologies that can work in a more energy efficient and environmentally sustainable way. Membrane processing may be one of the most promising solutions to the water crisis due to its high performance and low energy consumption. The study of new membrane materials, such as aquaporin (AQP) [1–4], carbon nanotubes (CNTs) [5, 6], and graphene [7], to produce advanced membranes is fast developing. Because of its high water permeability and excellent rejection of solutes [1–4], AQP offers an exciting opportunity to assemble high-flux and energy-efficient biomimetic membranes. Each AQP protein folds into an hourglass-shaped structure where the six transmembrane spanning *α*-helix segments surround a central pore structure defined by the two opposing asparagine–proline–alanine motifs [8]. The unique structure of AQP facilitates the fast transportation of water molecules. The hourglass structure also leads to the molecular sieve effect and electrostatic repulsion, which makes the proteins exhibit excellent salt rejection.

In recent years, AQP incorporated biomimetic membranes have received growing attention for nanofiltration (NF), reverse osmosis (RO), and forward osmosis (FO) applications [9–17]. Kumar *et al* predicted that AQP embedded biomimetic membranes could potentially achieve a high water flux of about 600 L m^−2^ h^−1^ (LMH), which is about two orders of magnitude higher than that of commercial RO membranes [9]. Thereafter, many methods have been developed to prepare defect-free and stable biomimetic membranes. In previous studies, an AQP incorporated supported lipid bilayer (SLB) was deposited on a commercial NF membrane through the vesicle fusion method [10]. Then AQP incorporated ABA copolymer bilayers were prepared on methacrylate functionalized cellulose acetate membranes for NF applications [11]. More recently, several studies have shown that proteoliposomes or proteopolymersomes can be directly used to prepare biomimetic membranes. A high performance RO membrane was produced by an interfacial polymerization method to embed proteoliposomes into a polyamide layer [12]. A layer-by-layer assembly method was also utilized to adsorb poly-L-lysine covered proteoliposome molecules onto polyanion membranes [13]. Wang *et al* immobilized crosslinked proteopolymersomes onto supported membranes and then sealed them by a layer-by-layer polydopamine–histidine coating process [18]. Although considerable effort has been made to produce AQP embedded membranes, the AQP protein has shown lower permeability in biomimetic membranes than its theoretical values. More work is clearly needed to develop high performance and stable biomimetic membranes.

The carrier system has significant influence on the performance of AQP-based biomimetic membranes. One of the possible factors is the lipid mobility of the carrier membrane. It has been reported that low mobility could inhibit AQP’s function [14]. To investigate the effect of lipid mobility on AQP incorporated membranes, monoolein (MO) was added to the saturated phospholipid DPPC (1, 2-dipalmitoyl-sn-glycero-3-phosphocholine) and unsaturated phospholipid DOPC (1,2-dioleoyl-sn-glycero-3-phosphocholine) SLBs. MO is a kind of non-toxic and biocompatible monoglyceride, which possesses a negative spontaneous curvature and can thus assemble into non-lamellar phases, such as bicontinuous cubic and inverted hexagonal phases [19]. The incorporation of MO into saturated phospholipid (DPPC) bilayers increases the molecular mobility in the bilayers because of the additional free volume induced by the *cis*-double bond of MO.

In this study, NTR7450 and NF270 supported DPPC, DPPC/MO, DOPC, and DOPC/MO SLBs were prepared as carrier membranes with different fluidities. AQP1 was used as the water channel protein to reveal the performance of the composite SLBs in NF membranes. Through permeability and salt rejection measurements, the effect of MO on the AQP1 incorporated phospholipid membranes was systematically studied.

## 2. Materials and methods

### 2.1. Materials

1,2-dioleoyl-sn-glycero-3-phosphocholine (DOPC, purity >99%), 1,2-dipalmitoyl-sn-glycero-3-phosphocholine (DPPC, purity >99%), and 1,2-dipalmitoyl-sn-glycero-3-phosphoethanolamine-N-(7-nitro-2-1,3-benzoxadiazol-4-yl), ammonium salt (NBD-PE, purity >99%) were purchased from Avanti Polar Lipids (USA). Monoolein (MO, purity >95%) was kindly supplied by Danisco Cultor (Denmark). AQP1 was purchased from Cusabio Biotech Co., Ltd (China). 1-*n*-octyl-*β*-d-glucopyranoside (OG, purity >99%) was purchased from Sigma Aldrich. Other materials such as sodium chloride (purity >99%), monopotassium phosphate (purity >99%), dipotassium phosphate (purity >99%), were of the highest purity available. Milli-Q water (Millipore, integrated ultrapure water system) with a resistivity of 18.2 M*Ω* cm was used. Phosphate buffered solution with pH = 7.5 was prepared using 10 mM KH_2_PO_4_, 10 mM K_2_HPO_4_·3H_2_O and 150 mM NaCl. The ratios mentioned in the paper are in molar fraction unless otherwise stated. A flat sheet sample of the NF270 membrane (Dow-Filmtec) was provided by the manufacturers and the NTR7450 membrane was purchased from Hydranautics/Nitto Denko.

### 2.2. Preparation of liposomes

A certain amount of lipid was prepared by the evaporation of chloroform under a N_2_ stream from a chloroform solution of lipids. The dried lipid film was left to stand overnight in a vacuum to remove the residual solvent, and then rehydrated in phosphate buffer at a final concentration of 0.1 mg ml^−1^ through vortexing, followed by five freeze–thaw cycles. Uniform vesicles were obtained by extrusion (11 times through 100 nm polycarbonate membranes) through an extruder system purchased from Avanti Polar Lipids, Alabaster, AL, USA. For the DPPC/MO and DOPC/MO mixtures, samples with different MO molar ratios (*R*_MO_ = moles of MO/moles of phospholipids) were prepared at the same total lipid concentration of 0.1 mg ml^−1^.

### 2.3. Preparation of AQP1 incorporated liposomes

10 mM potassium phosphate buffer (pH = 7.5), 150 mM NaCl, 10% (v/v) glycerol, 1% (w/v) OG and AQP1 were mixed together to obtained a 2 × 10^−3^ mg ml^−1^ AQP1 solution.

A certain amount of the AQP1 solution was added into four kinds of liposomes (DPPC, DOPC, DPPC/MO and DOPC/MO) containing 1% OG with different protein to lipid ratios (*R*_AQP1_ = moles of AQP1/moles of MO and phospholipids). The solution was dialyzed in a 6–8 kDa molecular weight cut-off (MWCO) dialysis bag (Spectra Por) against 1 L phosphate buffer for 3 days at room temperature. The buffer was refreshed every day. After dialysis, the proteoliposome solution was extruded through a polycarbonate membrane with a mean pore size of 100 nm for further use.

### 2.4. Preparation of NF membrane substrates

NF membranes were soaked in a 50% ethanol/water (v/v) solution for 10 min and then washed for 5 min in Milli-Q water. Before use, the NF membranes were stored in the Milli-Q water for over 24 h.

### 2.5. Formation of SLBs on NF membranes

Deposition of the supported lipid layer was carried out by the vesicle fusion method facilitated by the hydraulic pressure on the NF membrane surface. Phospholipid liposomes were spread onto NF270 and NTR7450 membranes and equilibrated at room temperature for 2 h. In order to improve the mechanical strength of the supported lipid membranes for water flux and rejection measurements, all liposomes were spread on the NF membrane for 1 h under −0.1 MPa pressure. The membrane was rinsed several times to remove the residual dissolved lipids.

### 2.6. Membrane characterization

Atomic force microscopy (AFM) measurements were performed on a Multimode AFM with a Nanoscope V MultiMode controller (Veeco, USA) using manufacturer supplied software. Tapping mode measurements in liquid were performed using NP-S cantilevers (short lever, nominal spring constant 0.06 N m^−1^). All AFM images were obtained by *in situ* measurements. The morphology of the surface was obtained from topography scans using the instrument software.

Fourier transform infrared (FTIR) spectra were recorded in the attenuated total refection (ATR) mode using a Bruker-Tensor 27 FTIR spectrometer (Bruker, Germany) to characterize the presence of functionalized groups in the lipid (DPPC, DOPC, DPPC/MO, DOPC/MO) structure and the NF membranes. Each spectrum was recorded over the range 500–4000 cm^−1^.

An electrokinetic analyzer (Anton Paar SurPASS, Austria) was used to measure the membrane potential based on streaming potential measurements wherein 1 mmol L^−1^ KCl was used as electrolyte solution at pH 7.5.

Fluorescence images and fluorescence recovery after photo-bleaching (FRAP) were obtained in a LSM 510 Meta system (Carl Zeiss, Germany) with a 20× objective under 488 nm excitation. For fluorescence characterization, lipids were mixed with 0.5% NBD-PE (0.5 wt%) in chloroform before liposome preparation. FRAP was performed by bleaching a 10 *μ*m diameter spot under the same conditions. 488 nm excitation light was used for FRAP bleaching at 100% intensity to obtain images at 10% intensity. All fluorescence experiments were performed *in situ*. The diffusion coefficient was calculated using the following equation: 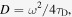 where *D* is the diffusion coefficient, *ω* is the diameter of the photo-bleached spot and *τ*_D_ is the characteristic time of diffusion [20].

The hydrophilicity of the NF membranes and SLBs was characterized via static contact angle measurements. Prior to contact angle measurement, all the membranes were vacuum-dried overnight. A DSA 100 system (Krüss, Germany) was used to perform the measurements. This device was equipped with a high speed video camera to monitor the side images of the drop profile. Milli-Q water (2 *μ*l) was injected on the membrane surface using a microsyringe under ambient conditions. The contact angle was then measured after 10 s for the membranes and an average value was obtained by seven measurements for each membrane.

### 2.7. NF performance of membranes

The water flux and salt rejection of each membrane were measured by a homemade cross-flow NF device. The effective area of the membrane was 1956 mm^2^. The flow rate was 40 l h^−1^, and the velocity was 20 cm s^−1^. The measurements were performed in a stainless steel cell for about one hour by using 2 g l^−1^ NaCl solution under the conditions of 4 bars and room temperature. The permeation flux (*J*_V_) was determined at least 1 h after the start of the NF tests to allow for stabilization. *J*_V_ was calculated from equation (1):

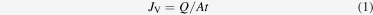
where *Q* is the increase of permeate volume (l) over a certain period of time *t* (h), and *A* is the effective filtration area. The salt rejection (*R*) was calculated according to equation (2):

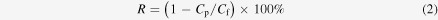
where *C*_p_ and *C*_f_ are the concentration of the permeate solution and the feed solution, respectively.

## 3. Results and discussion

### 3.1. Characterization of the NF and SLB membranes

#### 3.1.1. Morphologies by in situ AFM

**Figure 1.**
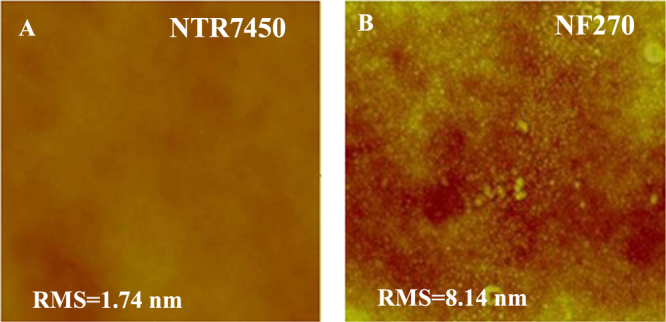
*In situ* AFM images (scan size 5 × 5 *μ*m^2^, *z*-scale 500 nm) of (A) NTR7450 and (B) NF270.

Figure 1 presents the surface morphologies of NTR7450 and NF270. Both NTR7450 and NF270 exhibit compact NF membrane surfaces. NTR7450 exhibits a very flat surface with a low root mean square roughness (RMS) value of 1.74 nm, but NF270 shows a rougher surface with an RMS of 8.14 nm [21].

**Figure 2.**
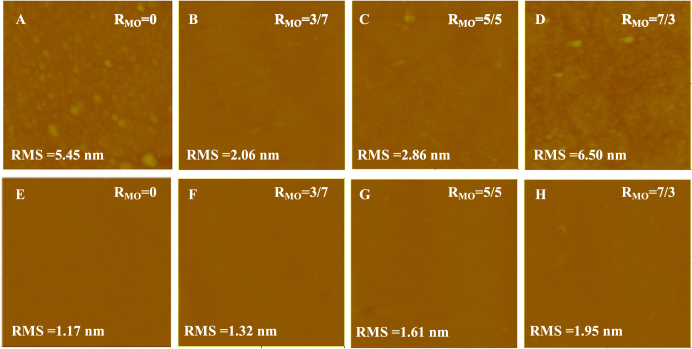
*In situ* AFM images (scan size 5 × 5 *μ*m^2^, *z*-scale 500 nm) of DPPC/MO vesicles deposited on NTR7450 surface with different *R*_MO_ values, (A) *R*_MO_ = 0, (B) *R*_MO_ = 3/7, (C) *R*_MO_ = 5/5 and (D) *R*_MO_ = 7/3; DOPC/MO vesicles deposited on NTR7450 surface with different *R*_MO_, (E) *R*_MO_ = 0, (F) *R*_MO_ = 3/7, (G) *R*_MO_ = 5/5 and (H) *R*_MO_ = 7/3.

As shown in figure 2, the influence of MO on the formation of SLBs on the NTR7450 surface was investigated by *in situ* AFM. For pure DPPC (*R*_MO_ = 0, figure 2(A)), we can see many bright spots with an RMS of 5.45 nm. These may be induced by the existence of intact vesicles on the NTR7450 surface. As the experimental temperature (25 ± 1 °C) is lower than the phase transition temperature of DPPC (41 ± 1 °C), the DPPC vesicles are in gel phase and possess high bending rigidity. Therefore, the intact vesicles or vesicle patches would be adsorbed on the NF surface [22]. With the increase in *R*_MO_ (*R*_MO_ = 3/7 and *R*_MO_ = 5/5), SLBs can be clearly observed in figures 2(B) and (C), accompanied by the decrease of the RMS value. This reveals that the addition of an appropriate amount of MO is favorable for the formation of the DPPC/MO SLB at room temperature which is in accordance with previous reports that the *gauche* structure of MO’s *cis*-double bond significantly disrupts the tight and efficient packing of DPPC molecules in bilayers, reduces the phase transition temperature of DPPC from gel to liquid crystal, and enhances the molecular mobility [22, 23]. These factors are hence favorable for the formation of SLBs. However, as shown in figure 2(D), greater MO component (*R*_MO_ = 7/3) induces the appearance of some bright spots on the substrate surface and a high RMS value of 6.50 nm. A possible reason is that the large amount of MO in the DPPC/MO system prefers to assemble in a cubic liquid crystal phase, which is stiffer than the vesicles and hinders the formation of an SLB.

The effect of MO on the formation of DOPC SLBs on the NTR7450 surface is illustrated in figures 2(E)–(H). A flat DOPC/MO SLB can be observed except for a few defects in figure 2(H). As DOPC exhibits a lower phase transition temperature (−18 ± 1 °C) than DPPC [24, 25], DOPC vesicles are in the liquid crystal phase. It has been reported that liquid crystal phase phospholipid vesicles can form an SLB with few defects [26–28]. The DOPC SLB, in contrast to the DPPC layer, shows a low RMS value. In addition, MO has similar unsaturated chain structures to DOPC, which makes MO have a minor effect on the formation of a high quality DOPC SLB. Moreover, as a result of the appearance of the cubic liquid crystal phase formed by MO, the RMS value increases with the increase of *R*_MO_ in the DOPC/MO SLB membranes.

**Figure 3.**
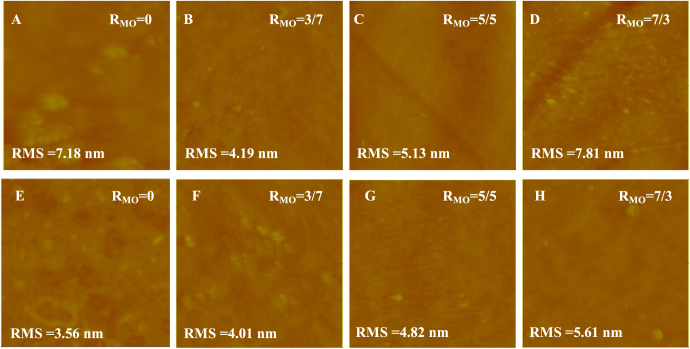
*In situ* AFM images (scan size 5 × 5 *μ*m^2^, *z*-scale 500 nm) of DPPC/MO vesicles deposited on NF270 surface with different *R*_MO_ values, (A) *R*_MO_ = 0, (B) *R*_MO_ = 3/7, (C) *R*_MO_ = 5/5 and (D) *R*_MO_ = 7/3; DOPC/MO vesicles deposited on NF270 surface with different *R*_MO_, (E) *R*_MO_ = 0, (F) *R*_MO_ = 3/7, (G) *R*_MO_ = 5/5 and (H) *R*_MO_ = 7/3.

AFM images of the NF270 supported lipid membranes are shown in figure 3. More high spots or patches can be observed in the NF270 supported systems compared with the NTR7450 SLB membranes. These may be induced by the higher roughness of NF270 compared to NTR7450 [29], which inhibits the formation of a defect-free SLB [14]. Another reasonable explanation is that lipids prefer to adhere to NTR7450 rather than NF270, because of the higher dissociation of the sulfonic groups of NTR7450 compared to the carboxylic groups of NF270 [21].

#### 3.1.2. ATR-FTIR and zeta potential

**Figure 4.**
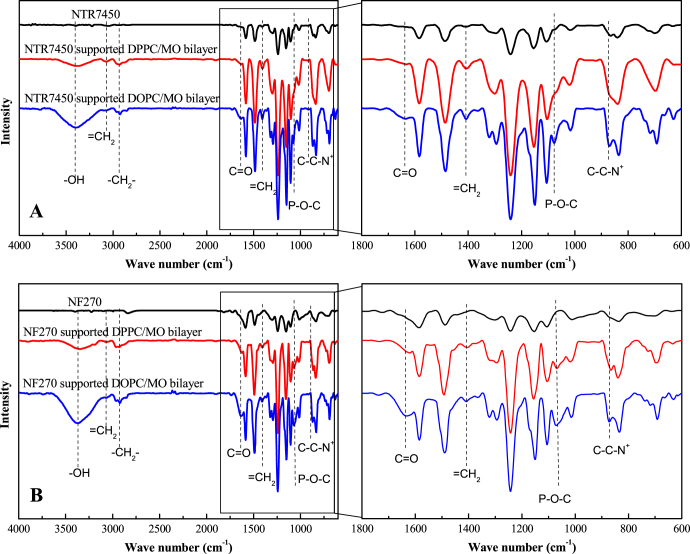
FTIR spectra of NF membrane supported lipid membranes. Top: NTR7450 SLBs (*R*_MO_ = 5/5) membrane (A); bottom: NF270 SLBs (*R*_MO_ = 5/5) membrane (B).

ATR-FTIR was used to confirm the formation of SLBs on the NF substrates. From figure 4, we can observe the 1303/1240 cm^−1^ doublet peaks and the band at 1151 cm^−1^, which correspond to asymmetric stretching and symmetric stretching of O=S=O groups of NTR7450 [30]. For NF270, the N–H in-plane bending vibration peak at 1580 cm^−1^ and the C–N stretching vibration corresponding to a shoulder peak at 1438 cm^−1^ can be observed [30]. DPPC/MO (*R*_MO_ = 5/5) and DOPC/MO (*R*_MO_ = 5/5) SLBs have the same characteristic peaks. The band at 3480 cm^−1^ is attributed to hydroxyl stretching of MO’s glycerin group [31]. The peaks at 2923 cm^−1^ and 1637 cm^−1^ are methylene stretching of the mixed lipids’ aliphatic tails and the hydrogen-bonded carbonyl groups, respectively [31, 32]. Two new peaks appear at 1068 cm^−1^ and 900 cm^−1^ after SLB formation, which are associated with the P–O–C and C–C–N^+^ groups of phospholipid hydrophilic heads [33]. These results confirm the existence of lipid mixtures on both NTR7450 and NF270 surfaces.

Table 1 summarizes the zeta potential data of the prepared membranes. Both NF270 and NTR7450 exhibit negative surface charges [21]. Because of the formation of SLBs or adsorbed vesicle layers on the NF surfaces, the zeta potential increases. This can be attributed to the lipid coverage of the negatively charged surface. Interestingly, the zeta potential of DPPC/MO SLB on either NTR7450 or NF270 is higher than that of the DPPC layers, which indicates that an SLB membrane with fewer defects is formed by the addition of MO to DPPC. However, MO plays a different role in the DOPC/MO SLB as observed in AFM analysis. The DOPC/MO SLBs exhibit decreased zeta potential compared with the DOPC SLBs, suggesting that more defects are induced by the incorporation of MO in the DOPC SLB.

**Table 1.** Zeta potential of various membranes (pH = 7.5, 1 mM KCl).

Membranes	NTR7450 (mV)	NF270 (mV)
NF membrane	−51	−58
DPPC (*R*_MO_ = 0)	−36	−41
DPPC/MO (*R*_MO_ = 5/5)	−30	−29
DOPC (*R*_MO_ = 0)	−22	−28
DOPC/MO (*R*_MO_ = 5/5)	−37	−44

#### 3.1.3. Fluorescence microscopy and FRAP investigations

**Figure 5.**
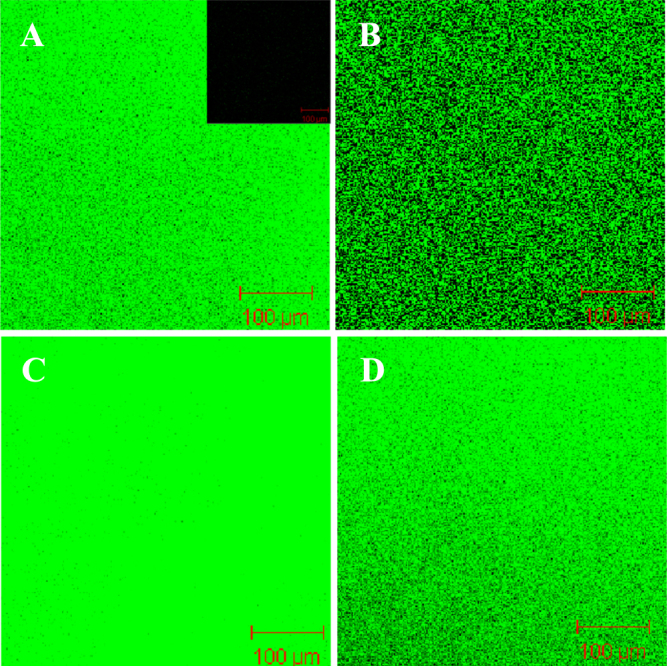
Confocal fluorescence images of NTR7450 supported DPPC/MO and DOPC/MO bilayers with 0.5 wt% NBD-PE. (A) DPPC (*R*_MO_ = 0), (B) DPPC/MO (*R*_MO_ = 5/5), (C) DOPC (*R*_MO_ = 0), (D) DOPC/MO (*R*_MO_ = 5/5). Inset of (A) is the fluorescence image of virgin NTR7450.

**Figure 6.**
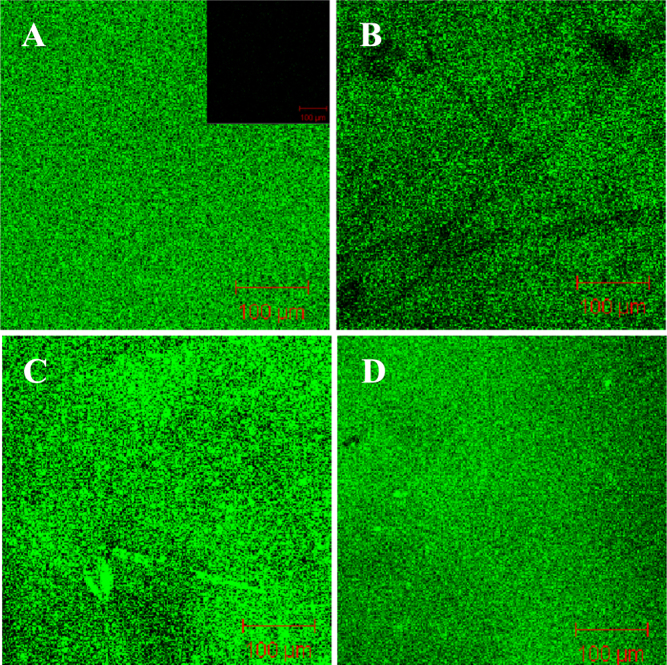
Confocal fluorescence images of NF270 supported DPPC/MO and DOPC/MO bilayers with 0.5 wt% NBD-PE. (A) DPPC (*R*_MO_ = 0), (B) DPPC/MO (*R*_MO_ = 5/5), (C) DOPC (*R*_MO_ = 0), (D) DOPC/MO (*R*_MO_ = 5/5). Inset of (A) is the fluorescence image of virgin NF270.

To better observe the formation of SLBs on NF membranes, a fluorescent membrane probe, NBD-PE (0.5 wt%), was spiked into the liposomal vesicles before the formation of the complex, and then the resultant material was subjected to confocal laser scanning fluorescence microscopic observation. As shown in figures 5 and 6, no fluorescence signal was detected in NTR7450 and NF270 (insets of figures 5(A) and 6(A)). After formation of the SLB, uniformly distributed fluorescence signals from the NBD-PE probe can be found for the NTR7450 and NF270 supported DPPC, DPPC/MO (*R*_MO_ = 5/5), DOPC, and DOPC/MO (*R*_MO_ = 5/5) bilayers. This phenomenon demonstrates the formation of lipid membranes on the NF substrates.

**Figure 7.**
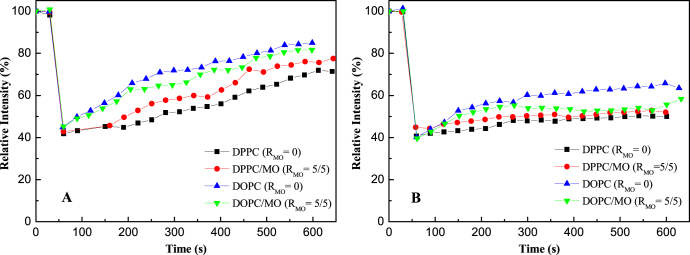
The FRAP curves of NTR7450 (A) and NF270 (B) SLB membranes.

Lipid mobility plays an important role in the performance of the incorporated AQP in the SLB membranes [10, 14, 21, 34]. FRAP was used to examine the lipid mobility in the SLB membranes. The recovery of fluorescence within the photobleached region is due to random motion or diffusion of the unbleached fluorescent lipid molecules from the surroundings over time. FRAP profiles of the NTR7450 and NF270 supported lipid membranes are shown in figure 7. The fluorescence intensities of the bleached regions decline to ∼40% for all membranes. After 600 s, various recovery percentages can be observed. The diffusion coefficient (*D*) of lipids in the membranes is roughly calculated to be in the range of ∼10^−13^ to ∼10^−12^ m^2^ s^−1^, which is lower than the lipid mobility in the cell membrane (∼10^−10^ to ∼10^−11^ m^2^ s^−1^) and in the SLB on glass or mica (∼10^−10^ m^2^ s^−1^) [10, 14]. This may be attributed to the rough surface of the NF membranes. As can be deduced from figure 7, the lipid mobility increases in the sequence DPPC < DPPC/MO (*R*_MO_ = 5/5) < DOPC/MO (*R*_MO_ = 5/5) < DOPC on both NTR7450 and NF270 substrates. This indicates that MO’s *cis*-unsaturated chain provides greater free volume for molecular motion. Thus, the tightly packed straight-chain DPPC bilayer is disturbed, which makes the hydrophobic interior of the DPPC bilayer more fluid. The higher molecular mobility facilitates the reorganization and rupture of the adsorbed vesicles and then the formation of SLBs. While DOPC has the same hydrocarbon chain structure as MO, it is believed that MO does not increase the lipid mobility of the DOPC SLB. However, the formation of stiff MO cubic liquid crystals hinders the rapid diffusion of DOPC molecules. Then, the fluorescence recovery of the DOPC SLB is faster than that of the DOPC/MO SLB.

Also of note, NF270 supported membranes (figure 7(B)) show slower recovery than NTR-7450 supported membranes (figure 7(A)). This may be caused by the higher roughness of NF270 compared to NTR7450.

### 3.2. NF performance

#### 3.2.1. Contact angle measurements

**Figure 8.**
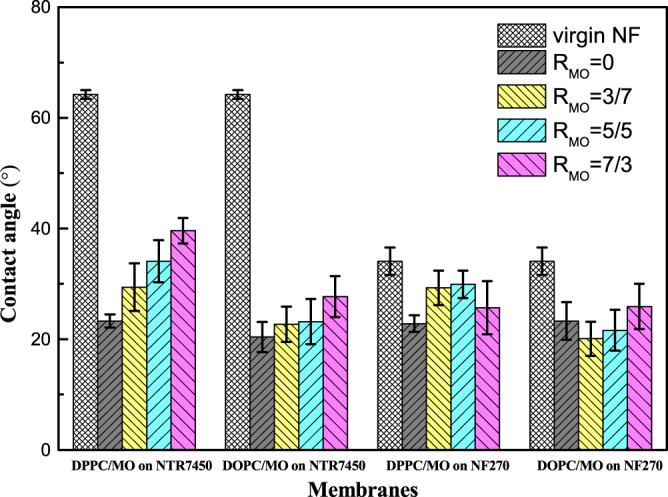
Contact angles of NTR7450 and NF270 SLB membranes. Each point represents the mean ± standard deviation of three samples.

As shown in figure 8, the surface hydrophilicity of the SLB is also influenced by the addition of MO. The contact angles of virgin NTR7450 and NF270 are 64.2° and 34.1°, respectively. The formation of an SLB decreases the contact angles of the composite membranes, which suggests an increase in hydrophilicity. The contact angles of membranes covered with DPPC and DOPC bilayers are about 23°, which is consistent with previous studies [35]. With the increase in *R*_MO_, the contact angle of the composite membranes slightly increases, because the glyceryl group of MO is less hydrophilic than the phosphocholine group of the phospholipids. Besides, the stiff cubic phase of MO on the NF membrane surface inhibits the formation of a continuous SLB, which results in a decrease in the hydrophilicity [36].

#### 3.2.2. Pure water flux

**Figure 9.**
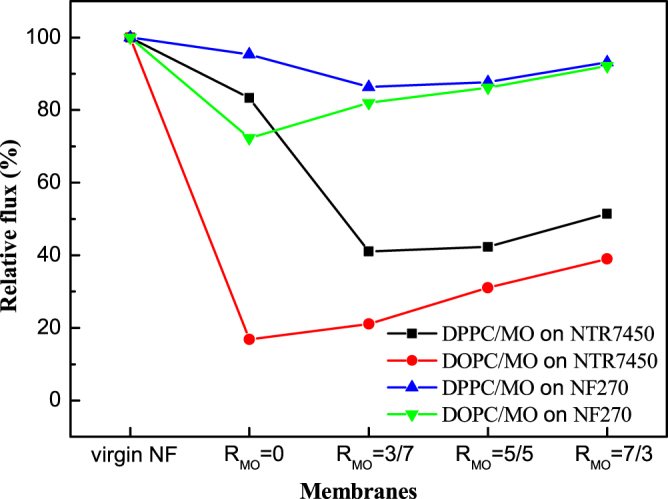
Effect of different content of MO SLBs on the relative pure water flux of SLB membranes.

The pure water flux data are listed in table 2. The pure water flux values of NTR7450 and NF270 are 34.8 and 101.2 LMH, respectively, which correlate well with previously reported values [37]. To quantitatively assess the coverage of the SLBs on the NF membranes, the relative pure water flux is calculated (relative flux = pure water flux of the SLB covered NF membranes/pure water flux of the substrate NF membrane ×100%). As shown in figure 9, the relative flux of the NTR7450 supported DPPC membrane (*R*_MO_ = 0) is 83%. This suggests that there are many defects in the DPPC layer. Then, water and solutes can penetrate the composite membranes through the defects. As discussed above, many intact DPPC vesicles are deposited on the NTR7450 surface and hinder the formation of a continuous SLB [38]. Meanwhile, for DPPC/MO SLB membranes, the relative flux decreases to ∼40% (*R*_MO_ = 3/7 and *R*_MO_ = 5/5). This indicates the formation of a better SLB than pure DPPC. However, a greater MO component (*R*_MO_ = 7/3) in the binary system tends to form some cubic phase defects and then the relative flux increases to 51%. NF270 supported DPPC/MO membranes show similar curves, but the relative flux is much higher than for NTR7450 supported membranes. This may be caused by the efficient self-assembly of a homogenous SLB on NTR7450, because of its lower roughness and more highly charged sulfonic groups compared to NF270 [10, 21].

**Table 2.** Pure water flux (LMH) of NF SLBs membranes.

	DPPC/MO on NTR7450	DOPC/MO on NTR7450	DPPC/MO on NF270	DOPC/MO on NF270
Virgin NF	34.8 ± 1.4	34.8 ± 1.4	101.2 ± 4.6	101.2 ± 4.6
*R*_MO_ = 0	29.0 ± 0.7	5.8 ± 0.4	96.5 ± 4.5	73.2 ± 2.1
*R*_MO_ = 3/7	13.5 ± 0.7	7.3 ± 0.5	87.4 ± 3.0	82.9 ± 2.8
*R*_MO_ = 5/5	14.0 ± 0.7	10.8 ± 0.6	88.8 ± 2.8	87.3 ± 3.1
*R*_MO_ = 7/3	16.5 ± 1.3	13.5 ± 0.5	94.3 ± 3.8	93.3 ± 3.9

Each point represents the mean ±standard deviation of three samples.

The DOPC SLB on NTR7450 shows the lowest relative flux of 17%, which suggests that NTR7450 is well covered by the DOPC SLB. The relative flux shows a significant increase when more MO is introduced into the DOPC bilayer, because MO induces some defects in the DOPC/MO SLBs and leads to an increase of the relative flux.

#### 3.2.3. Performance of the AQP1 incorporated SLB membranes

**Figure 10.**
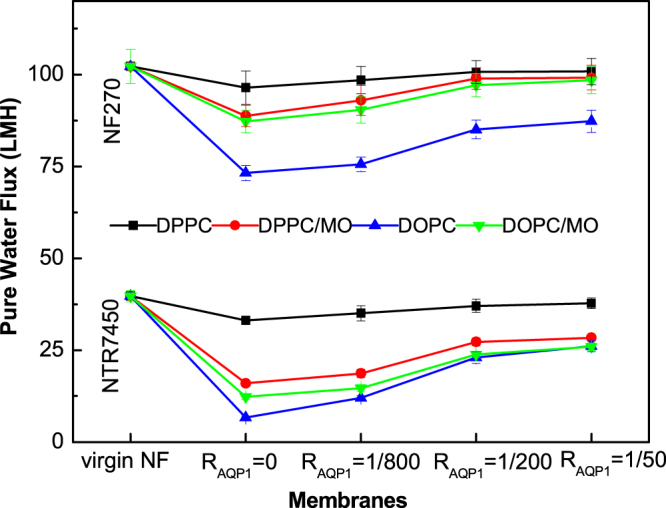
Pure water flux of NTR7450 and NF270 supported DPPC, DPPC/MO (*R*_MO_ = 5/5), DOPC, and DOPC/MO (*R*_MO_ = 5/5) bilayer membranes with different *R*_AQP1_.

Mixed lipid bilayers were prepared as carrier membranes to incorporate the water channel protein (AQP1). Figure 10 shows the pure water fluxes of AQP1 incorporated biomimetic membranes. The permeability of the AQP1 incorporated SLBs on both NF substrates show a similar trend. At the same *R*_AQP1_, the pure water flux decreases in the sequence DPPC > DPPC/MO (*R*_MO_ = 5/5) > DOPC/MO (*R*_MO_ = 5/5) > DOPC. This sequence is consistent with the lipid mobility and SLB quality, which can be understood from the different effects of MO on DPPC and DOPC bilayers. It is noteworthy that these high fluxes are caused by defects rather than the permeable nature of AQP1. Besides, the NF270 supported biomimetic membranes possess more defects, therefore the pure water fluxes of the NF270 supported membranes are higher than those of the NTR7450 supported ones at the same *R*_AQP1_.

The permeability enhancement induced by the incorporation of AQP1 can be deduced from the pure water flux increment compared with the AQP1 free membranes (table 3). The increment increases with the incorporation of more AQP1, which is consistent with previous reports that SLBs were used directly as selective layers [11, 21]. However, it was also reported that a further increase in the AQP molar ratio (*R*_AQP_ ≥ 1/50) leads to a decline in the permeability for polymer sealed proteoliposome or proteopolymersome membranes [18, 21, 39], which may be caused by increased resistance in the polymer selection layer.

**Table 3.** Pure water flux increments (LMH) induced by the incorporation of AQP1.

	NTR7450 supported membranes			NF270 supported membranes		
	*R*_AQP1_ = 1/800	*R*_AQP1_ = 1/200	*R*_AQP1_ = 1/50	*R*_AQP1_ = 1/800	*R*_AQP1_ = 1/200	*R*_AQP1_ = 1/50
DPPC	1.7	3.5	4.1	2.0	4.2	4.3
DPPC/MO	2.3	9.9	10.8	4.1	10.0	10.4
DOPC	4.7	14.3	17.0	2.4	11.8	14.1
DOPC/MO	2.1	10.1	11.9	3.1	10.2	11.3

At the same *R*_AQP1_, the increment of pure water flux increases in the sequence DPPC < DPPC/MO (*R*_MO_ = 5/5) < DOPC/MO (*R*_MO_ = 5/5) < DOPC, which is in good agreement with the lipid mobility sequence as aforementioned. In a sense, higher lipid mobility may facilitate water molecule transportation through APQ1. Because MO increases the mobility of molecules in the mixed lipid bilayers, the permeability of AQP1 is enhanced in the DPPC/MO SLB. In contrast, the addition of MO lowers the pure water flux increment of the AQP1 incorporated DOPC SLB.

**Figure 11.**
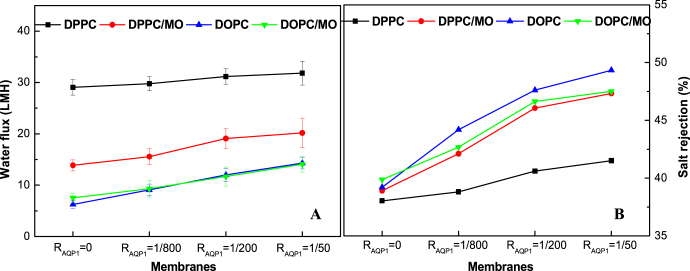
Water flux (A) and NaCl rejection (B) of NTR7450 supported DPPC, DPPC/MO (*R*_MO_ = 5/5), DOPC, and DOPC/MO (*R*_MO_ = 5/5) bilayer membranes with different *R*_AQP1_.

Figure 11 shows the permeability and NaCl rejections of the NTR7450 supported biomimetic membranes. A similar trend can be observed for the permeability with pure water flux. The NaCl rejection of AQP1 incorporated DPPC, DPPC/MO, DOPC, DOPC/MO membranes (*R*_AQP1_ = 1/50) is 41.5%, 47.3%, 49.3%, and 47.5%, respectively. It is speculated that the addition of an appropriate amount of MO improves both the quality of the SLB and the mobility of molecules in the mixed lipid bilayers, which is beneficial for the permeability and salt rejection of the AQP1 incorporated DPPC biomimetic membranes. However, MO has a negative impact on the AQP1 incorporated DOPC SLB because of the formation of a cubic liquid crystal phase.

**Figure 12.**
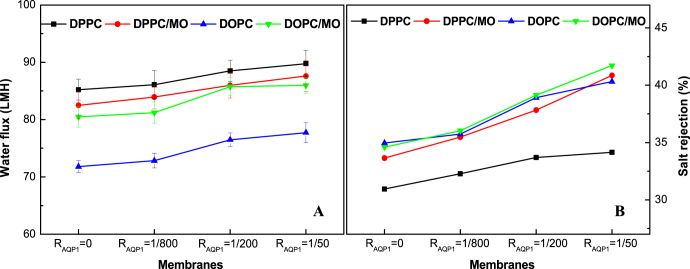
Water flux (A) and NaCl rejection (B) of NF270 supported DPPC, DPPC/MO (*R*_MO_ = 5/5), DOPC, and DOPC/MO (*R*_MO_ = 5/5) bilayer membranes with different *R*_AQP1_.

The fluxes and NaCl rejections of the NF270 supported biomimetic membranes are presented in figure 12. Both the water flux and NaCl rejection increase with the incorporation of more AQP1. Although the water fluxes are higher than those of NTR7450 supported biomimetic membranes, the NaCl rejections are much lower because of the existence of many defects in the NF270 supported lipid membranes [14, 21].

## 4. Conclusions

We prepared and characterized AQP1 incorporated biomimetic membranes by adding MO to tune the lipid mobility and quality of biomimetic membranes. Addition of MO (*R*_MO_ = 5/5) to saturated DPPC promotes the formation of a high mobility SLB membrane with fewer defects. MO in DPPC bilayers decreases the phase transition temperature of the DPPC/MO system and enhances the lipid mobility because of the *cis*-double bond of MO. While DOPC and MO have the same hydrocarbon chain structure, the addition of MO does not improve the lipid mobility of the DOPC/MO system. Moreover, MO cubic liquid crystals in the DOPC SLB inhibit lipid motion and induce some defects.

The permeability and salt rejection of the AQP1 incorporated composite membranes are measured by the cross-flow method. The augmentation of *R*_AQP1_ in the NF supported membranes improves both the flux and NaCl rejection. MO changes the mobility and quality of the phospholipid bilayers, both of which are reported to be closely related to the performance of AQP1 incorporated SLBs. The lipid mobility increases in the sequence DPPC < DPPC/MO (*R*_MO_ = 5/5) < DOPC/MO (*R*_MO_ = 5/5) < DOPC. The flux increment and salt rejection of the AQP1 embedded biomimetic membranes increase with lipid mobility. Although the permeability and salt rejection are not significantly improved, this report provides some new insight into the fabrication of AQP incorporated biomimetic membranes. In future work, the MO additive membrane system will be further developed to obtain defect-free biomimetic membranes for water purification and desalination.
